# Exogenously applied silver nanoparticles (AgNPs) differentially affect bacterial blight disease control in twenty-seven wheat cultivars

**DOI:** 10.1186/s12870-024-05424-7

**Published:** 2024-07-23

**Authors:** Seyyedeh Zahra Fatemifard, Asad Masoumiasl, Bahman Fazeli-Nasab, Ramin Piri, Ali Reza Mirzaei, Ali Salehi Sardoei, Mansour Ghorbanpour

**Affiliations:** 1 Department of Agronomy and Plant Breeding, Agriculture Faculty, Yasouj University, Yasouj, Iran; 2 Department of Agronomy and Plant Breeding, Agriculture Institute, Research Institute of Zabol, Zabol, Iran; 3 Department of Agronomy and Plant Breeding, Agriculture Faculty, University of Tehran, Tehran, Iran; 4 Department of Agronomy and Plant Breeding, Faculty of Agriculture and Natural Resources, University of Mohaghegh Ardabili, Ardabil, Iran; 5 Department of Horticulture, Faculty of Environmental and Fisheries Sciences, Gorgan University of Agricultural Sciences and Natural Resources, Gorgan, Iran; 6 Department of Medicinal Plants, Faculty of Agriculture and Natural Resources, Arak University, Arak, 38156-8-8349 Iran; 7 Institute of Nanoscience and Nanotechnology, Arak University, Arak, 38156-8-8349 Iran

**Keywords:** Antioxidant system, Bacterial blight, Nano silver, Plant protection, Resistant cultivars, Wheat

## Abstract

The bacterial blight of wheat is an important global disease causing a significant decline in crop yield. Nanotechnology offers a potential solution for managing plant diseases. Therefore, this research aimed to investigate the effectiveness of silver nanoparticles (AgNPs) in controlling bacterial blight in 27 locally grown wheat cultivars. The study examined the impact of AgNPs at three distinct time points: 1, 3, and 5 days after the onset of the disease. Biochemical assay revealed that one day after applying the disease stress, the Inia cultivar had the highest amount of soluble protein (55.60 μg.g^−1^FW) content in the treatment without AgNPs. The Azadi cultivar, without AgNPs treatment, had the lowest amount of soluble protein content (15.71 μg.g^−1^FW). The Tabasi cultivar had the highest activity of the superoxide dismutase (SOD) (61.62 mM.g^−1^FW) with the combination treatment of AgNPs. On the other hand, the Karchia cultivar had the lowest SOD activity (0.6 mM.g^−1^FW) in the treatment of disease without AgNPs. Furthermore, three days after the application of stress, the Mahdavi cultivar had the highest amount of soluble protein content (54.16 μg.g^−1^FW) in the treatment of disease without AgNPs. The Niknejad cultivar had the highest activity of the SOD (74.15 mM.g^−1^FW) with the combined treatment of the disease without AgNPs. The Kavir cultivar had the lowest SOD activity (1.95 mM.g^−1^FW) and the lowest peroxidase (POX) activity (0.241 mM g^−1^FW min^−1^) in the treatment of the disease with AgNPs. Five days after exposure to stress, the Mahooti cultivar had the highest SOD activity (88.12 mM.g^−1^FW) with the combined treatment of the disease with AgNPs, and the Karchia cultivar had the lowest SOD activity (2.39 mM.g^−1^FW) in the treatment of the disease with AgNPs. Further, the results indicated that exposure to AgNPs could improve the antioxidant properties of wheat seeds in blight-infected and disease-free conditions in some cultivars.

## Introduction

In Iran, a significant portion of the country’s land is used for rainfed farming, providing a substantial amount of the country’s food production. Dry grains, particularly wheat, play a crucial role in this agricultural landscape [1, 2]. Bacterial blight is a notable disease affecting wheat, caused by the bacterium *Pseudomonas syringae* pv. *syringae*. This disease results in reduced plant height, grain yield, and overall growth of wheat plants. It is a widespread issue globally, causing extensive damage to wheat fields annually. Challenges in controlling this disease include the inefficient use of certain antibiotics and the development of resistance and stability to some copper compounds in the environment [3].

Nanotechnology is a highly significant technology worldwide, with distinct characteristics and a wide range of applications in various scientific and technological fields. Nanoparticles are atomic or molecular complexes with dimensions ranging from 1 to 100 nm, and they possess unique physical and chemical properties compared to their bulk materials [4–6]. Nanoparticles have made widespread impact due to their unique importance and characteristics [7–9]. Depending on their size, nanoparticles can easily cross cell organelles through the plasma wall and membrane, affecting a series of metabolic processes [10–12]. Nanotechnology has provided an opportunity to enhance the capabilities of natural antioxidant enzymes through the use of nanozymes, offering a new solution to address their existing limitations.

Nanotechnology in agriculture aims to improve plant disease resistance and enhance nutrient utilization for plant growth. Nanoparticle technology is important in addressing agricultural issues related to plant-pathogen interaction and developing innovative methods for product protection. Agricultural chemicals are currently applied to crops through spraying, but nano agrochemicals must be designed with specific properties, including effective concentration, high solubility, stability, efficacy, controlled release mechanisms, enhanced targeted activities, and lower toxicity levels for safe delivery. Silver has various effects on living organisms, and it can protect plants from fungal, viral, and microbial diseases depending on its concentration and forms. The introduction of AgNPs has been found to improve root length growth in crops such as california poppy (*Eschscholzia californica* Cham [13], geranium (*Pelargonium zonale*) cultivars [14–17]. In a different study, the growth of rice seedlings was further enhanced by AgNPs. This was shown by a decrease in reactive oxygen species (ROS) levels, lipid peroxidation, catalase (CAT), superoxide dismutase (SOD), and H_2_O_2_ content compared to the control [18]. Plants with higher levels of antioxidants demonstrate greater resistance to oxidative damage. Both catalase (CAT) and peroxidase (POX) are crucial antioxidants that break down H_2_O_2_ into water and oxygen molecule [19, 20]. Plant defense against pathogens depends on structural defense characteristics and the expression of resistance-related genes. The plant’s ability to succeed in this aspect relies on the activity of its antioxidant system and the concentration of reactive oxygen species. The plant can lower the concentration of reactive oxygen species and reduce their harmful effects using enzymatic and non-enzymatic mechanisms [21–23].

The antioxidant defense system includes enzymes such as SOD, CAT, guaiacol peroxidase, and ascorbate peroxidase. It also includes non-enzymatic antioxidants such as flavonoids, anthocyanins, and other phenolic compounds, as well as ascorbate, alpha-tocopherol, and beta-carotene [24, 25]. There have been limited studies on how AgNPs can improve the physiological characteristics of wheat seedlings infected with bacterial blight. Therefore, it is necessary to conduct research in this area to identify wheat varieties resistant to the disease. The purpose of this research is to provide other researchers with information on the use of AgNPs treatments for managing bacterial blight disease in different wheat cultivars.

## Materials and methods

### Collecting and planting seeds

The seeds of 27 Iranian bread wheat cultivars (listed in Table 1) were obtained from the Karaj Seedling and Seed Breeding Institute in Karaj, Alborz Province. The study was conducted in January 2021 at the central laboratory of the Faculty of Agriculture, Yasouj University, using a completely randomized design (CRD) with three replications. Each pot (3 L) contained five wheat seeds of each cultivar and was filled with a mixture of autoclaved and sieved soil, consisting of agricultural soil (50%), animal manure (30%), and sand (20%). After two weeks of germination, three plants were maintained in each pot, and they were kept in controlled moisture, temperature (25°C), and lighting conditions (16 h of light and 8 h of darkness). A suspension was prepared and subjected to a 24-h zigzag cultivation process. After 24 h, the bacterial colonies were removed and transferred into Erlenmeyer flasks containing water. The bacterial suspension’s density was adjusted to an optical absorption rate of 0.5 (10^7^ colonies per ml) at a 600 nm optical wavelength using a spectrophotometer. Samples were collected at the 4–6 leaf stage and at 1, 3, and 5 days after inoculation. Each plant was cut from the soil surface, wrapped in aluminum foil, and immediately placed in a dry ice tank, and then transferred to a -40°C freezer. The data were analyzed using ANOVA and the mean square was calculated using the Tukey test at 1% and 5% levels of significance.

**Table 1:** The list of the studied Iranian native wheat cultivars

Cultivar Name	Reaction of living and non-living stresses
Azadi	Relatively resistant to yellow rust and semi-resistant to semi-sensitive to brown black rust, resistant to Russian aphid
Alamut	Resistant to yellow rust and sensitive to brown rust (10), semi-resistant to Russian aphid, semi-resistant to wheat spike septoriosis
Alvand	Relatively resistant to yellow rust and relatively sensitive to brown rust in some areas, resistant to Russian aphid, resistant to hidden black fungus
Atrak	Resistant to stem rust, semi-resistant to wheat spike septoriosis
Omid	Relative resistance to Russian aphid
Aynia	Good bakery quality
Bezostaya	Susceptible to wheat spike septoriosis
Boolani	Susceptible to hidden blackworm, susceptible to Russian aphid, susceptible to brown rust, susceptible to wheat powdery mildew
Tajan	Resistance to yellow rust, brown rust and Fusarium cluster, sensitive to latent black
Chamran	Susceptible to hidden blackworm, fully susceptible to wheat spike septoriosis
Darab2	Very sensitive to black leaf spot
Roshan	Salt tolerant, drought tolerant
Zarin	Resistant to yellow rust and sensitive to brown rust, resistant to hidden black, resistant to Russian aphid, sensitive to wheat powdery mildew
Sabalan	Resistant to yellow rust and sensitive to black spot
Sorkh-Tokhm	Semi-resistant to Russian aphid, sensitive to wheat powdery mildew
Sardari	Susceptible to Russian aphid
Sholeh	Susceptible to Russian aphid
Tabasi	Resistant to Russian aphid
Falat	Very sensitive to black leaf, sensitive to yellow rust and fusarium, semi-resistant to septoriosis, resistant to black rust
Quds	Susceptible to yellow rust, resistant to wheat spike septoriosis
Karchia	Salt tolerant
Kavir	Semi-resistant to black leaf spot, resistant to Russian aphid, sensitive to wheat spike septoriosis
Golestan	Semi-sensitive to all types of rust, resistant to black rust
Mahouti	Salt tolerant
Mahdavi	Resistant to Russian aphid
Mihan	Cold resistant, relative resistance to drought stress
NikNejad	In black rust disease, it is resistant to TRTFC race and sensitive to others

### Preparation of AgNPs solution and bacterial culture

In the present study, we obtained silver nanoparticles (AgNPs) from Spadan Company in Isfahan, Iran (https://product.statnano.com/company/spadan-green-giti-coating-co.). The experiment involved testing six different concentrations of AgNPs (1, 5, 50, 100, 200, 400 ppm) along with four types of antibiotic discs (amoxicillin, erythromycin, tetracycline, and penicillin) in three replications. To carry out the experiment, we cultured bacteria on agar medium for 24 h and then transferred a single colony onto fresh agar using a loop. Subsequently, antibiotic discs and nanoparticle-coated discs were placed onto the agar in three replicates in separate petri dishes at equal intervals using sterilized tweezers. The petri dishes were then placed in the incubator for 24–48 h. After the bacteria had fully grown in the petri dishes, we measured the halos around the discs. To ensure accuracy, the experiment was repeated a second time.

### Preparation of bacterial suspension

**Fig. 1:**
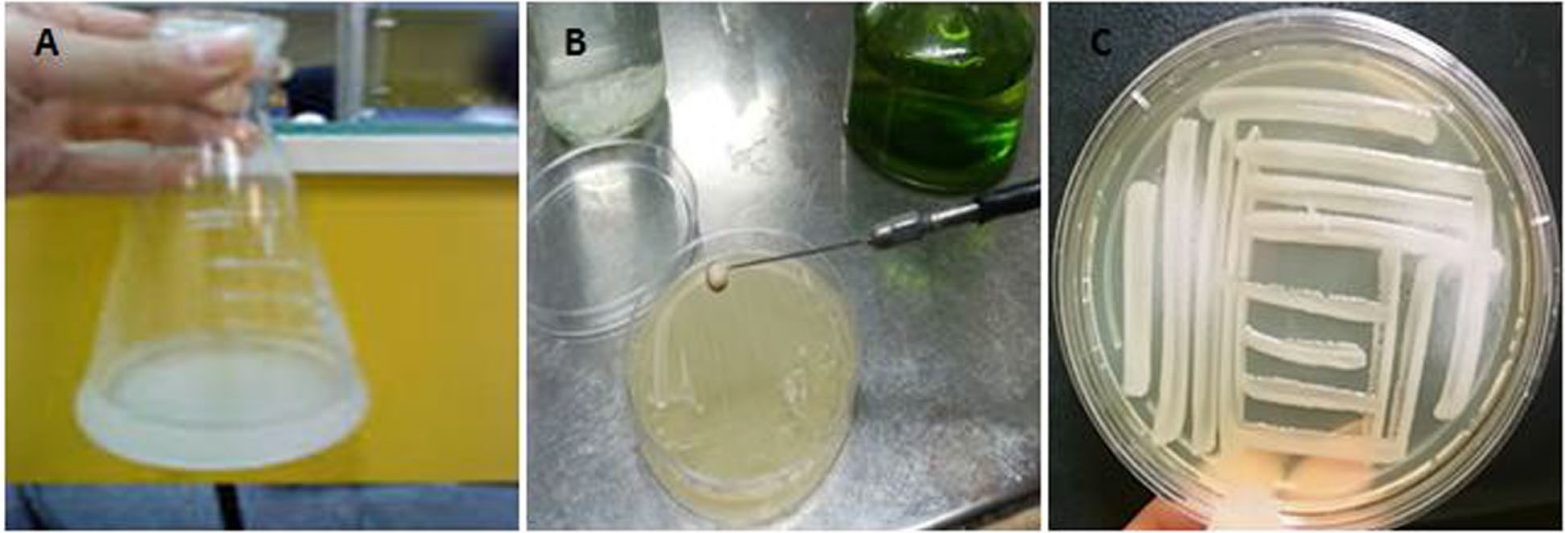
Preparation of suspension (**a**), isolate culture (**b**), and bacterial colony growth (**c**)

The following procedure was carried out: a beaker of autoclaved distilled water was placed under the hood for foliar spraying. After 24 h of growth, the cultured bacteria were removed from the surface of the culture medium using a loop and transferred to an Erlenmeyer flask. The spectrophotometer was zeroed using distilled water and then the absorbance of bacteria in the solution was read as 0.5 at a wavelength of 600 nm (Fig. 1). After preparing the bacterial suspension, foliar spraying was executed on 12/9/2021 at the 4–6 leaf stage. Each pot received a 25 ml spray of bacterial suspension. The control plants also received a 25 ml spray of autoclaved distilled water.

**Fig. 2:**
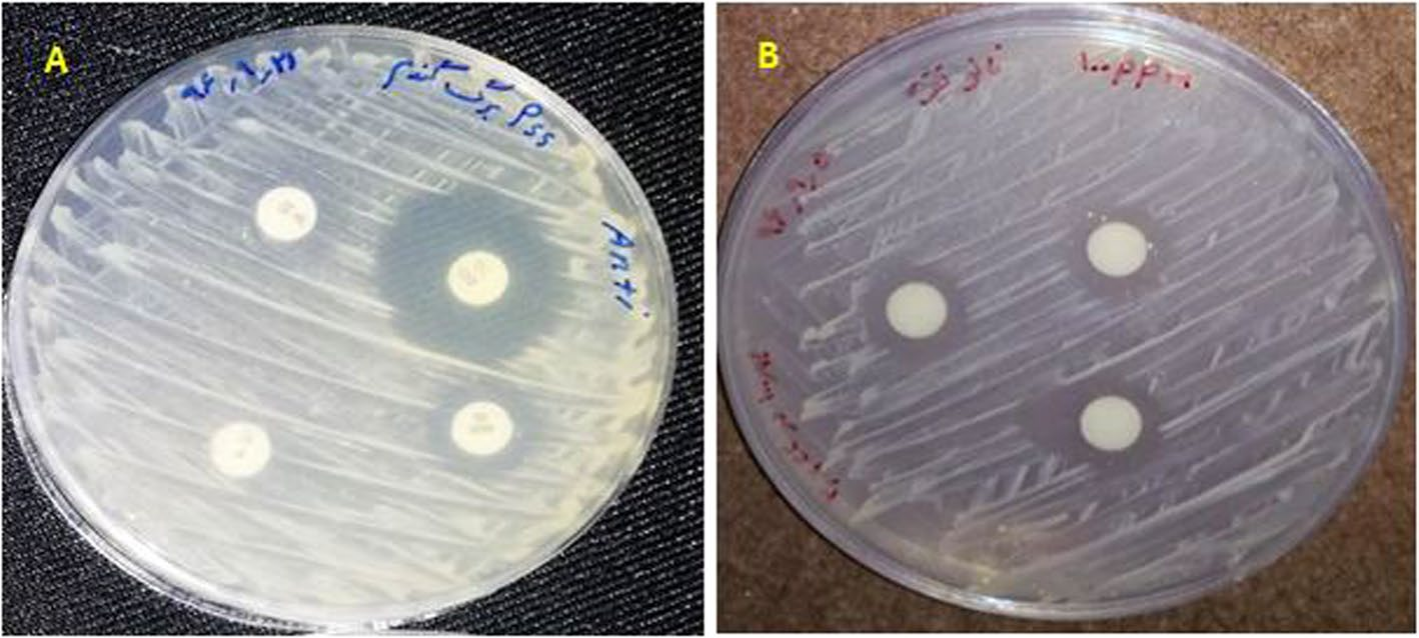
It shows the effect of AgNPs with diffusion disk (**a**) and the effect of the antibiotic disk (amoxicillin, tetracycline, erythromycin, and penicillin) on Pseudomonas bacteria (**b**)

Inoculation with the bacterial blight agent of wheat was performed when the wheat plants were at the 4–6 leaf stage [26–28]. Laboratory investigations [26–28] revealed that at concentrations of 200 ppm and higher, the antibacterial effect of silver nanoparticles (AgNPs) was even greater than that of antibiotics for all bacteria except Agrobacterium. The results showed that increasing the concentration of AgNPs led to a stronger antibacterial effect, equaling or surpassing the inhibitory effects of antibiotics. Consequently, AgNPs at a concentration of 100 ppm was selected and prepared. After 24 h of inoculation with the wheat bacterial blight agent, AgNPs were applied via foliar spraying at a rate of 25 ml per plant. Subsequent sampling was carried out at different time intervals (1, 3, and 5 days after inducing disease stress by spraying the bacterial solution on the leaves) (Fig. 2, A and B).

To check enzyme traits in periods of 1, 3, and 5 days after inoculation, leaf samples were collected, and to measure some biochemical traits, they were transferred to -40°Celsius refrigerator in a chamber containing dry ice.

### Quantitative measurement of leaf soluble protein

Bradford’s [29] method was used to measure the amount of protein. Bradford’s method is based on the binding of Coomassie Brilliant Blue 250 to protein in acidic medium and determination of maximum absorption from 465 to 595 nm. The absorbance at 595 nm is directly proportional to the protein concentration.

### Measuring the activity of POX enzyme

Peroxidase activity assay was performed by Panda et al. [30]. Briefly, 25 μl of the extraction supernatant was added to 1.95 ml 0.1 M phosphate buffer (pH 7.0), 40 mM H_2_O_2_ and 1.6% guaiacol containing the substrate buffer and spectrophotometric measurements were taken at 470 nm wavelength.

### Measurement of SOD enzyme activity

The superoxide dismutase (SOD, EC 1.15.1.1) enzyme activity was estimated by measuring the decrease in absorbance of formazone formed by the superoxide radical and nitro-blue tetrazolium (NBT) dye by the enzyme [31].

### Statistical analysis

The ANOVA was performed using Statistic ver 10 software. All figures were generated using Excel software. Means square was calculated using the Duncan test at 1 and 5 probability levels.

## Results

The variance analysis of the data revealed significant main effects of cultivars and the combined treatment of AgNPs and disease, as well as their interaction, on the activity of SOD and POX enzymes at the time of harvesting 1 day after applying stress, at a 1% probability level. Additionally, the main effects and their interaction on soluble protein were found to be significant at 1% and 5% probability levels, respectively (Table 2). Furthermore, at 3 and 5 days after stress, the main effects of the Nano + Disease treatment and cultivars, as well as their interaction, were significant at a 1% probability level for soluble protein levels and the activity of SOD enzyme. Additionally, the main effects and their interaction on POX were significant at the 1% and 5% probability levels (Tables 3 and 4).

**Table 2:** Analysis of variance (mean square) for the effect of AgNPs + Disease treatment and Cultivars on biochemical characteristics of wheat (Harvest time 1)

S.O.V	df	Soluble protein content	SOD activity	POX activity
Cultivar	26	198.20**	684.7**	0.0417**
Treatment	2	5339.6**	639.1**	0.9832**
Cultivar × Treatment	52	105.89*	278.3**	0.0329**
Error	162	64.67	17.16	0.0157
C.V. (%)	-	13.44	15.12	18.32

^*^ and ** are significant at *p* ≤ 0.05 and *p* ≤ 0.01 levels, respectively

**Table 3:** Analysis of variance (mean square) for the effect of AgNPs + Disease treatment and Cultivars on biochemical characteristics of wheat (Harvest time 2)

S.O.V	df	Soluble protein content	SOD activity	POX activity
Cultivar	26	142.1^**^	773.7**	0.0391^**^
Treatment	2	4790^**^	1429**	0.7985^**^
Cultivar × Treatment	52	106.3^**^	572.3**	0.0235v
Error	162	23.40	15.93	0.0147
C.V. (%)	-	11.63	13.82	12.55

^*^ and ** are significant at *p* ≤ 0.05 and *p* ≤ 0.01 levels, respectively

**Table 4:** Analysis of variance (mean square) for the effect of AgNPs + Disease treatment and Cultivars on biochemical characteristics of wheat (Harvest time 3)

S.O.V	df	Soluble protein content	SOD activity	POX activity
Cultivar	26	230.42**	976.7**	0.0392**
Treatment	2	5812.1**	597.3**	2.4548**
Cultivar × Treatment	52	274.07**	474.6**	0.0280*
Error	162	37.31	17.35	0.0173
C.V. (%)	-	9.48	12.73	17.38

^*^ and ** are significant at *p* ≤ 0.05 and *p* ≤ 0.01 levels, respectively

### Harvest time 1 day after stress

**Fig. 3:**
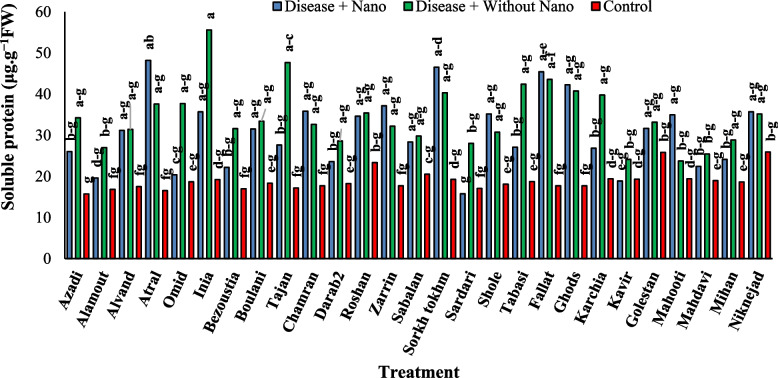
The effect of AgNPs + Disease combined treatment on the amount of soluble protein in 1 day after the application of disease stress in different wheat cultivars

**Fig. 4:**
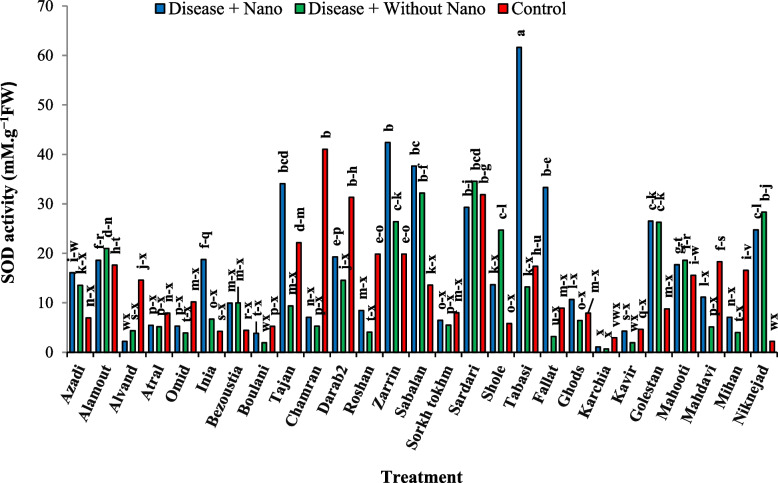
The effect of AgNPs + Disease combined treatment on the activity of SOD enzyme in 1 day after the application of disease stress in different wheat cultivars

**Fig. 5:**
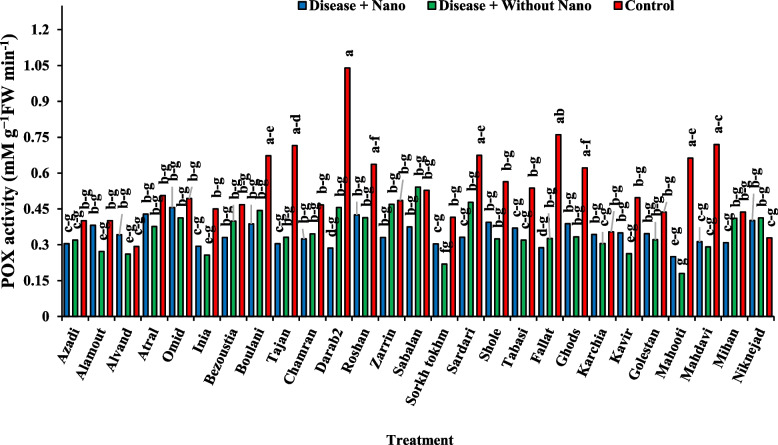
The effect of AgNPs + Disease combined treatment on POX enzyme activity 1 day after applying disease stress in different wheat cultivars

**Fig. 6:**
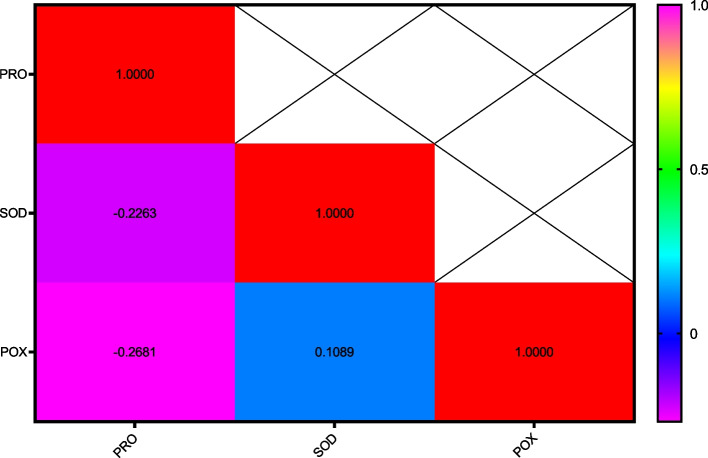
The correlation coefficients variance the effect of AgNPs + Disease treatment and Cultivars on some biochemical characteristics of wheat (Harvest time 1)

After one day of applying stress, the comparison of average data showed that the highest amount of soluble protein content in the disease treatment without AgNPs was found in the Inia cultivar, while the lowest amount was associated with the Kavir and Mahooti cultivars (Fig. 3). Additionally, the highest activity of the SOD enzyme was observed in the Tabasi cultivar with the combined treatment of AgNPs + disease, while the lowest level was related to the treatment of the disease without AgNPs in the Karchia cultivar (Fig. 4). The highest activity of the POX enzyme was seen in the Darab2 cultivar under disease-free conditions and without the use of AgNPs. The lowest activity of the POX enzyme was obtained from the disease treatment without AgNPs in the Mahdavi cultivar (Fig. 5). In Harvest time 1, the most negative correlation was found between POX and soluble protein content, followed by SOD and soluble protein content. The correlation between SOD and POX was not significant (Fig. 6).

### Harvest time 3 days after stress

**Fig. 7:**
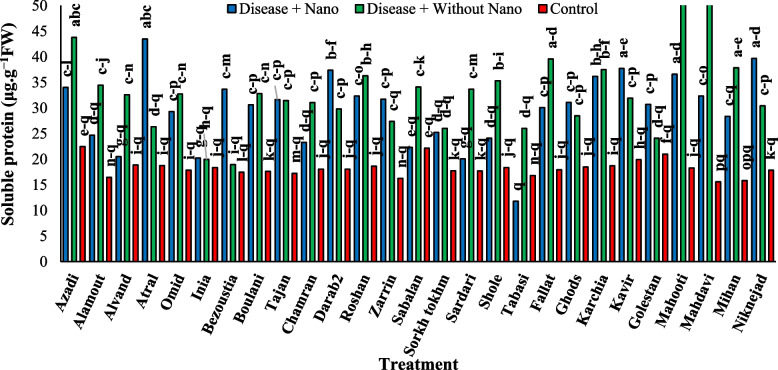
The effect of AgNPs + Disease combined treatment on the amount of soluble protein in 3 days after applying disease stress in different wheat cultivars

**Fig. 8:**
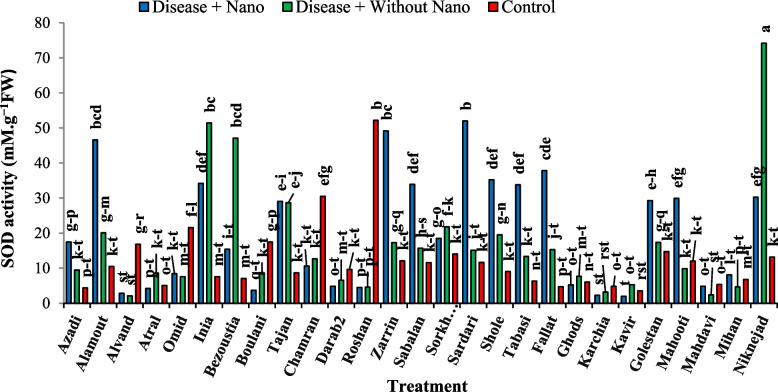
The effect of AgNPs + Disease combined treatment on the activity of SOD enzyme in 3 days after the application of disease stress in different wheat cultivars

**Fig. 9:**
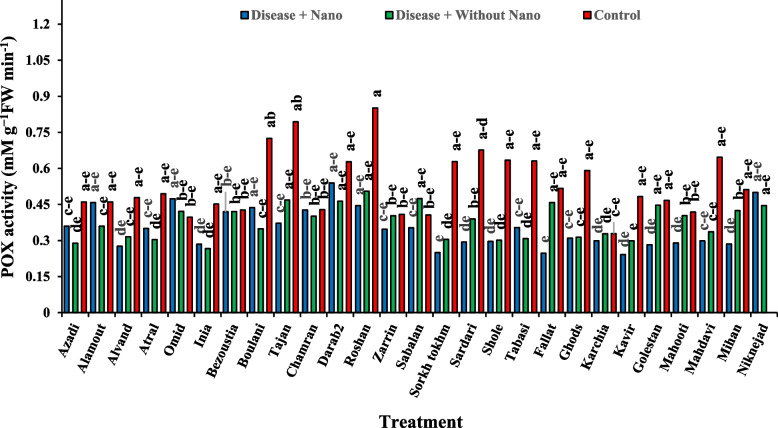
The effect of AgNPs + Disease combined treatment on POX enzyme activity in 3 days after applying disease stress in different wheat cultivars

**Fig. 10:**
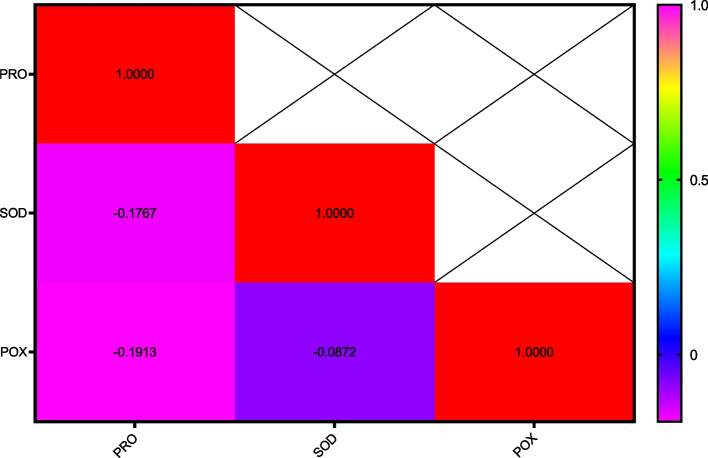
The correlation coefficients variance the effect of AgNPs + Disease treatment and Cultivars on some biochemical characteristics of wheat (Harvest time 3)

After three days of stress application, the Mahdavi cultivar showed the highest amount of soluble protein in the treatment without AgNPs, while the Tabasi cultivar had the lowest amount in the treatment with AgNPs (Fig. 7). Additionally, the Niknejad cultivar exhibited the highest SOD enzyme activity in the treatment without AgNPs, while the Kavir cultivar showed the lowest activity (Fig. 8). Moreover, the Roshan cultivar had the highest POX enzyme activity in the control treatment, whereas the Kavir cultivar had the lowest activity in the treatment with AgNPs (Fig. 9). Furthermore, the most negative correlation in Harvest time 3 was found between the POX antioxidant enzyme and soluble protein, followed by the correlation between POX and soluble protein. It was noted that the correlation between SOD and POX was not significant (Fig. 10).

### Harvest time 5 days after stress

**Fig. 11:**
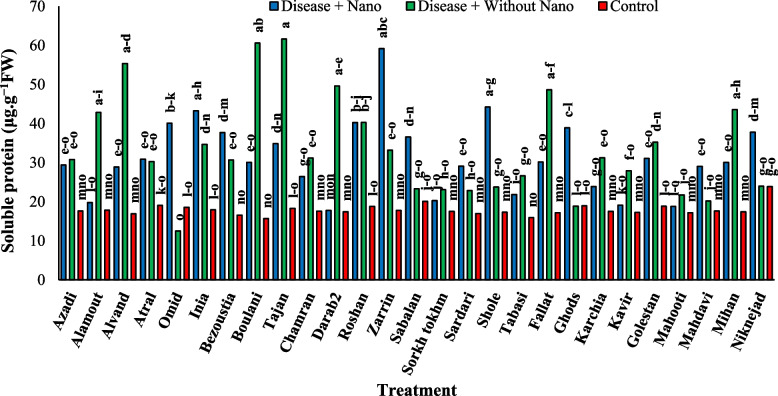
The effect of AgNPs + Disease combined treatment on the amount of soluble protein in 5 days after the application of disease stress in different wheat cultivars

**Fig. 12:**
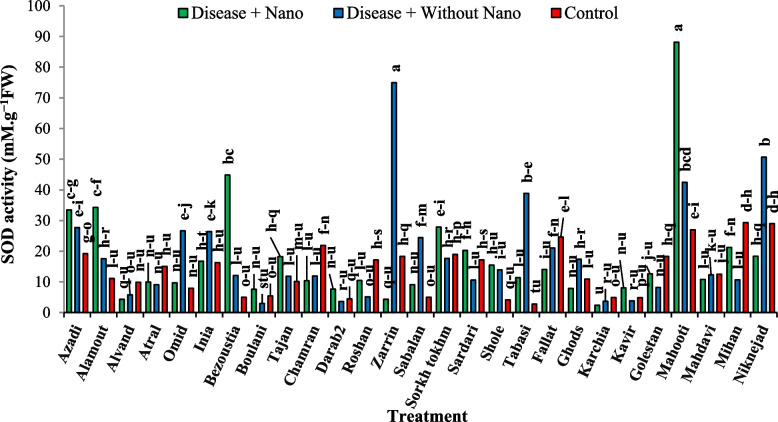
The effect of AgNPs + Disease combined treatment on the activity of SOD enzyme in 5 days after the application of disease stress in different wheat cultivars

**Fig. 13:**
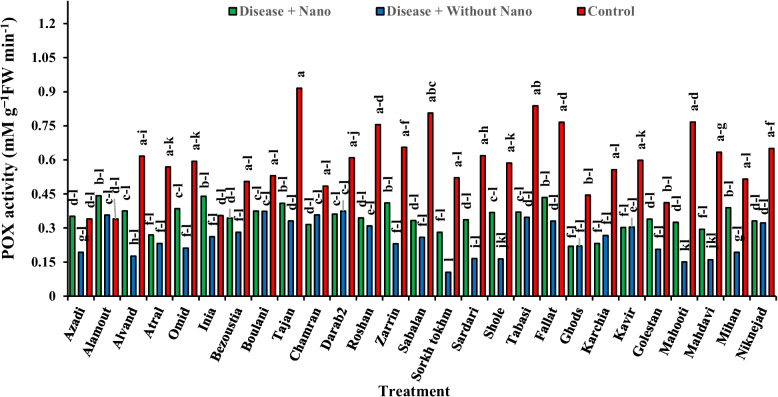
The effect of AgNPs + Disease combined treatment on POX enzyme activity in 5 days after applying disease stress in different wheat cultivars

**Fig. 14:**
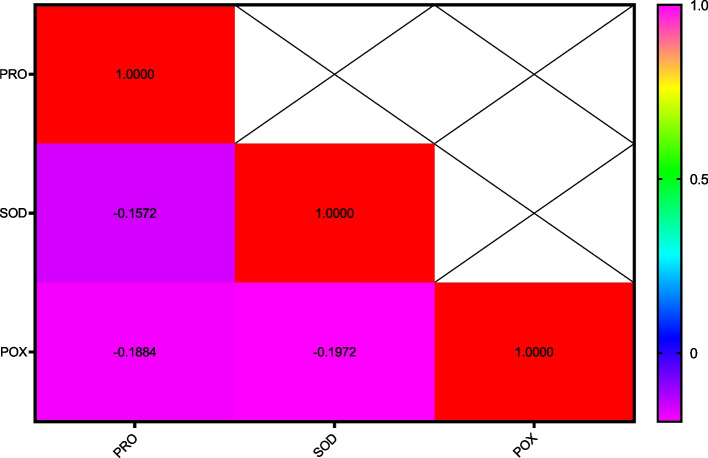
The correlation coefficients variance the effect of AgNPs + Disease treatment and Cultivars on some biochemical characteristics of wheat (Harvest time 5)

The analysis of the average data revealed that five days after stress application during harvesting, the Tajan cultivar showed the highest amount of soluble protein in the absence of AgNPs treatment for the disease. In contrast, the Tabasi cultivar had the lowest amount of soluble protein in the control treatment (Fig. 11). Additionally, the Mahooti cultivar exhibited the highest activity of the SOD enzyme in the combined treatment with AgNPs for the disease, while the Karchia cultivar had the lowest level of activity (Fig. 12). Furthermore, the Tajan cultivar in the control treatment demonstrated the highest activity of the POX enzyme, whereas the Sorkh Tokhm cultivar in the disease treatment with AgNPs showed the lowest level of activity (Fig. 13). Lastly, the most negative correlation at harvest time 5 was observed between soluble protein and the POX antioxidant enzyme, followed by the correlation between SOD and soluble protein (Fig. 14).

The analysis aimed to estimate variance components and genetic parameters of SOD, Soluble Protein, and POX traits in various wheat cultivars. The data analysis was conducted using the bounded maximum likelihood method in WOMBAT software. The genotypic and phenotypic variance of SOD, Soluble Protein, and POX traits were examined. In the POX trait, approximately 23.81% of the phenotypic variance was attributed to genetic variance, while for the Soluble Protein trait, this percentage was lower at about 55.43%. Based on these findings, it is recommended that genetic modifications should be prioritized for the POX trait, followed by the SOD trait, where 21.71% of the phenotypic variance is genetic (Table 5).

**Table 5:** Genotypic and phenotypic variances of Soluble Protein, SOD, and POX traits in the studied cultivars

Traits	Genetic Variance	Phenotype Variance	Genetic%
Soluble Protein	182.131	328.577	55.43023401
SOD	17,285	24,270.87	71.21705979
POD	0.0860284	0.1059036	81.23274374

## Discussion

Plants and plant cells have the ability to adapt to various conditions. When exposed to nanoparticles, plant cells may transfer chemicals to DNA, leading to changes in gene expression and affecting the plant’s growth and development. The impact of nanoparticles on plants depends on factors such as composition, concentration, size, and physical and chemical properties, as well as the type of plant species [32–35]. Bacterial infections such as blight and severe infections typically occur at the beginning of the growing season and during the plant’s growth stage. As the plant matures, it develops a type of resistance that protects it from disease damage. Developing resistant cultivars is one approach to reducing and controlling diseases. AgNPs have been successfully used as antifungal and antibacterial agents, offering a potential alternative to pesticides and chemical poisons. AgNPs exhibit enhanced antimicrobial properties due to changes in their physical and chemical properties when converted to nano dimensions, such as increased surface area [36, 37]. An important advantage of AgNPs is their impact on pathogenic microorganisms. Silver can react with the thiol group of microorganism enzymes, leading to enzyme denaturation and ultimately cell death [38].

The use of nanoparticle treatment has been found to increase the amount of soluble protein and the activity of antioxidant enzymes, which helps to control bacterial blight disease in wheat to some extent. The observed increase in enzyme activities may play a key role in protecting disease-infected plants against increased ROS production [39]. The increase in CAT enzyme activity in this research may be attributed to higher concentrations of AgNPs, which boost CAT activity to counter this compound [40]. Many researchers have reported the use of nano treatments to bolster plants’ defense systems against biotic and abiotic stresses. For instance, in a study on the treatment of *Hyoscyamus reticulatus* hairy roots with nano iron oxide stimulant, the highest CAT enzyme activity was achieved at a concentration of 900 mg L^−1^ over a 24-h duration [41]. Research has also shown that AgNPs and AgNO_3_ have varying effects on oxidative stress induction and the activity of antioxidant enzymes in *Allium cepa* roots [42], as well as in tobacco seedlings [43] and adult plants [44].

Living organisms have cell membranes that are negatively charged, while AgNPs are positively charged. The accumulation of positive charges of AgNPs on the negative charges of the cell membrane causes a change in the membrane structure. This leads to a loss of membrane permeability control, ultimately resulting in cell death [45]. In a separate experiment involving the medicinal plant buckwheat and different treatments of zinc nano oxide, it was observed that the activity level of a certain enzyme increased with concentrations above 100 mg/liter [46].

Exogenously applied AgNPs have displayed potential in managing bacterial blight disease in rice. Research has shown that AgNPs produced using *Bacillus cereus* SZT1 exhibited significant antibacterial activity against *Xanthomonas oryzae* pv. oryzae (Xoo), the bacteria responsible for causing bacterial leaf blight (BLB) disease in rice [47]. These nanoparticles were successful in inhibiting the occurrence of the disease and promoting plant growth, indicating their potential for controlling BLB [47]. Furthermore, AgNPs have a distinctive antibacterial mechanism that can help reduce the development of drug resistance in plant pathogens [48]. As for their impact on soluble protein and POX, the specific effects of externally applied AgNPs on these enzymes in the context of managing bacterial blight disease in rice are not explicitly mentioned in the search results provided. Further research may be necessary to determine the direct effects of AgNPs on these enzymes in the context of plant disease management [47, 48].

The use of nanoparticles for plant protection can occur through two different mechanisms. First, nanoparticles can be used to directly protect plants. Second, nanoparticles can serve as carriers for pesticides or other active substances, such as double-stranded RNA, which are applied by spraying, soaking seeds, leaf tissues, or roots [49–52]. Research [53] has investigated the inhibitory effect of AgNPs and copper nanoparticles on the growth of the fungus (*Wilsonomyces carpophilus*), which causes sieve spot disease in stone fruit trees. The study demonstrated that silver and copper nanoparticles at a concentration of 80 ppm had a significant difference compared to the two common fungicides, mancozeb and carbendazim. Furthermore, the investigation of the effect of AgNPs on the growth of the fungus *Rhizoctonia solani*, the cause of rice sheath blight, and the bacterium *Acidovorax avenae*, the cause of bacterial stripe spot disease in rice, reported that AgNPs have the potential to inhibit these plant diseases [53]. The amount of protein changes depending on the concentration of AgNPs used in the plant. Research has shown that silver-chitosan nanoparticles significantly increase the total protein in chickpea seedlings [54]. Another study observed an increase in the activity of CAT, guaiacol peroxidase, and ascorbate peroxidase in Brassica juncea due to the concentration of AgNPs [55]. Genetic and phenotypic diversity coefficients were used to estimate diversity, representing diversity in different traits influenced by genetic and environmental factors. In this study, the POX enzyme had the highest heritability, while soluble protein had the lowest. Heritability in all three traits was over 50%, but the genetic variance was lower than the phenotypic variance in all traits, indicating that environmental effects were greater than genetic influences.

## Conclusion

The findings indicate that the most effective approach to managing plant diseases involves utilizing nanotechnology, selecting specific cultivars, and conducting genetic investigations to identify resistant cultivars. The research suggests that environmental factors have a greater influence on these traits compared to genetic factors, as evidenced by the lower genetic variance relative to the phenotypic variance. As a result, it is advisable to prioritize traits influenced by genes when selecting resistant varieties and passing on this resistance to future generations. Furthermore, the study emphasizes the potential of using AgNPs as an effective method for combating plant diseases, offering a promising alternative to antibiotics and reducing their adverse impacts on organisms and ecosystems.

## Data Availability

All the data generated/analyzed during the study are available with the corresponding author on reasonable request.
